# Acknowledgement to Reviewers of *Medicina* in 2019

**DOI:** 10.3390/medicina56010041

**Published:** 2020-01-19

**Authors:** Medicina Editorial Office

**Affiliations:** MDPI, St. Alban-Anlage 66, 4052 Basel, Switzerland

The editorial team greatly appreciates the reviewers who have dedicated their considerable time and expertise to the journal’s rigorous editorial process over the past 12 months, regardless of whether the papers are finally published or not. In 2019, a total of 792 papers were published in the journal, with a median time to first decision of 35 days and a median time from submission to publication of 62 days. The editors would like to express their sincere gratitude to the following reviewers for their generous contribution in 2019:
Abdullah Al-ZaghalKiyoshi TakagiAbed KhalailehKlaus ButtenschoenAbheepsa MishraKlaus GratzAbhi PandhiKlaus-Peter DieckmannAbhishek MangaonkarKnud Bonnet YderstrædeAbram WagnerKok Yong ChinAbu Faem Mohammad Almas ChowdhuryKommidi HarikrishnaAdam Domonkos TarnokiKonstantinos MarkouAdam P. MortonKonstantinos PapazoglouAdam RaikesKooi Yepng KhawAdam ReichKosei NagataAdam T. EggebrechtKosuke YoshiharaAdela PenesovaKotaro TakedaAdeleye J. AfolayanKrebs MarkusAdérito SeixasKrister LindmarkAditi J. RavindranathKristina GorsetaAditya DandekarKristine RuppertAdriana DussoKrisztina M. Papp-WallaceAdriana NowakKriti PuriAdriana OlarKruthika SundaramAdriana V. GaitánKrzysztof BecAdriano LimaKuan-Fu ChenAdwitiya KarKumar VaibhavAgata Maria RabuazzoKurt NaberAgata StanekKurt WidhalmAgbangla Nounagnon FrutueuxKyle BurghardtÁgnes CzibulaKyle CaveAgostino BruzzoneKyriakos KachrimanisAhmed AbuzaidKyung Taek RimAida DuarteLars BrechtelAiping LiuLars KattnerAkira UmemuraLars P. KamolzAlasdair MacKenzieLaura GuidettiAlberto ElmiLaura KairevičėAlberto Marcos Heredia-RizoLaura OrsoliniAlejandra TomasLaura VicenteAleksandar VišnjićLaurent PlantierAleksi TornioLawrence C. MadoffAlena BukováLech SedlakAlene ToulanyLeigh FrameAlessandra FerramoscaLeila YoussefianAlessandra PelagalliLeopoldo Aguilera-AguirreAlessandro AlaimoLetty De WegerAlessandro ArenaLian-Hua HuangAlessandro CrisciLibor HejsekAlessandro De SireLina PadervinskienėAlessandro MalobertiLinas RovasAlessandro MatteLinda C. GiudiceAlessandro MoroLindsey GreviskesAlessandro ScanoLisa BossAlexander HemprichLisa CrawfordAlexander SeifertLisa Pescara-KovachAlexander V IvanovLiwei LangAlexandra TarabolettiLjubica DjukanovićAlexandros GeorgakilasLluisa QuevedoAlexandros MitsiosLongshuang HuangAlexandru CorlateanuLoo Keat WeiAlexandru Florin RogobeteLoredana PucaAlexey SarapultsevLorella PasquinucciAlfonso Rubio-NavarroLorenz TheilmannAlfred N. FontehLorenzo DesideriAli Borzabadi-FarahaniLorna DoyleAli ErsenLouise MarstonAli JanatiLourdes Cortes-DericksAli Salehzadeh-YazdiLuana Tenorio LopesAlicja M GruszkaĽubica ĎudákováAlina KuryłowiczLuc TimmermansAlina PoppLuca D’OnofrioAlison Sally PoultonLuca FiorilloAllan ColverLuca MorelliAllen L. HoLuca ProsperiniAllison B ReissLuci DusseAlphonse G. TaghianLucia GalvagniAmanda BrueglLucio MangoAmanda J. NicollLudmila BoublikovaAmanda N. Szabo-ReedLuigi Isaia LeccaAmaricai ElenaLuigi CormioAmerigo GiudiceLuis M. Pérez-BelmonteAmin KomeiliLuis Abel QuiñonesAmin R. JaverLuis Jara PalomaresAmit KumarLuís Pereira-da-SilvaAmit Kumar SahaLuis Rodrigo SaezAmosy E M’KomaLuiz André Freire PimentaAmr Abdulfattah Hausien HassanLuiza FrankAmrita DesaiLynda SpelmanAmrita SarkarLyudmila I. RachekAmritlal MandalM. UllmanAmutha RamadasMaciej Gawecki Amy MessersmithMaciej KurpiszAna Catarina QueirogaMaddalena De BernardoAna Filipa SilvaMadhulata Singh ChauhanAna Isabel EsquifinoMafalda LaranjoAna Isabel RibeiroMagali MillecampsAna M. L SecaMagdalena Hagner-DerengowskaAna M. PeiróMahendran ChinnappanAna PenezicMaija DambrovaAnandharajan RathinasabapathyMakoto HiraoAnandi LawMaksymilian ChruszczAnastasios KarayiannakisMalachi J. McKennaAnca Oana DoceaMałgorzata DołowyAnca Pantea StoianMalgorzata KosteckaAnders Christian LarsenMałgorzata OlszowyAndras BikovMalgorzata SyczewskaAndré Perez-PottiMallikarjun BidarimathAndrea CrespoManuel García-GoñiAndrea Crespo SedanoManabu MoriyaAndrea D. ThompsonManuela CabiatiAndrea SistiManuela Wally OssolaAndréa StinghenMara LeimanisAndrea TinnirelloMara SimopoulouAndrea TrevisanMarci SontagAndrea VianelloMarco CicciùAndreea SoareMarco FabbriAndrei SurguchovMarco Matteo CicconeAndrew C. TalkMarco PizzoferratoAndrew GrayMarco PortelliAndrew James WoodMaren GoeckenjanAndrew S. DayMargarete C. KulikAndric OrtizMaria Antonietta CiardielloAndrzej SurdackiMaria CappuccilliAngela McGlashanMaría Del Carmen Pérez-FuentesAngela SyMaria J. CrespoAngela YangMaría José García-BarradoAngelica GiulianiMaría José PenzolAngelija ValančiūtėMaria José Quina GaldinoAngelo DavalliMaria Lorenza MuiesanAngelo TroedhanMaria Maddalena AngioniAnil VermaMaria MasulliAnn L. GibsonMaria MesuracaAnna EsparhamMaria PerdomoAnna J. SchreinerMaria Rosário AlmeidaAnna Kucinska-ChahwanMaría Teresa Pérez-GraciaAnna ManolopoulosMaria-Fernanda Lorenzo-GómezAnna NordenströmMarian KlingerAnna PolewczykMarian VladescuAnna WardowskaMariangela De RobertisAnnelie PlentzMarianna BarbalinardoAnthony B. MillerMarianne ArnerAnthony MagitMariella PazzagliaAntoine LanotMarijana MarkovicAntonio Magán-FernándezMarilza Vieira Cunha RudgeAntonio MourinoMarina JerebtsovaAntonio SerranoMarina RagužAntonio Simone LaganàMario D. GarrettAntonio SteccoMario Damiano ToroAnubhab MukherjeeMario DioguardiAnusha PenumartiMario MacisArgyro MaziotiMario PretiAri ShechterMario PuvianiArif AsifMarius BirleaAristeidis H. KatsanosMarius MiglinasArnaud MachelartMariusz Da̧browskiArrigo CiceroMarjeta TerčeljArthur J. SiegelMark A. PerazellaArtur SłomkaMark Douglas BakerArun Kumar NayanataraMark E. WilliamsAsha KumariMark EckertAshkan ShoamaneshMark G JonesAshlesha KaushikMark M. WalshAshok PatilMarko PokornAthanasios PoulopoulosMarkus S. KupkaAtsushi KohgaMarta WanatAtul Kumar PandeyMartin BurtscherAudrey Franceschi BiagioniMartin DegenAuksė DomeikienėMartin ManningerAxel SeuserMartin O. SavageAzael J. Herrero Martin ŠimkovičBabken AsatryanMartina KroppBalaji OletyMartina MeszarosBaoyin ZhaoMartina ŠlajBárbara AngelMartina TomićBarbara NazareMarzena LaskowskaBarbara TomaselloMasaaki KurasakiBarbara VincensiMasaaki ShimataniBeata Morak-MłodawskaMasahiro IijimaBee Kang TanMasami KojimaBela FuelesdiMasataka SumiyoshiBen C. L. ChanMassimiliano CadamuroBenilde CosmiMassimiliano PolastriBeverley Jane HuntMassimiliano VanossiBharani Krishna Mynampati ArunadithyaMatteo GoldoniBharani SrinivasanMatteo RitrovatoBharat VaidyanathanMatthew ForemanBhesh Raj SharmaMatthias GatzBin HuMatthias KapischkeBisher H. AbuyassinMatthias SchefflerBłażej KudłakMatthias ZeilerBo Young HongMauro AlaibacBogdan Andrei SuciuMaxime PichonBojana M. Andrejić VisnjicMeera TandanBon Ham YipMehdi MenaiBongju KimMeik KunzBrandon G. RocqueMelinda HeinzBrenda DaviesMeng-Yu WuBrian D. PooleMeraj Alam KhanBrian PiperMichael Carlo GiovingoBrijesh Kumar SinghMichael EdelsteinBryan MarkinsonMichael FrassCamelia DiaconuMichael GramlichCamilla CallegariMichael JohnsonCarina TörnMichael KoenneckeCarla PiresMichael LangCarlo AlfieriMichael LavagninoCarlo ContiniMichael LichtenauerCarlo DiaferiaMichael S. KellyCarlo TicconiMichael TsatsosCarlos AyánMichal CiebieraCarlos Fernando MourãoMichał Jan StasiowskiCarlos Romero MoralesMichał KunickiCarlos RosadoMichal LatalskiCarmela CaputoMichal ŠteffCarmela SaturninoMichel HanssCarmelo Lucio SturialeMichela CasellaCarmen GherasimMichele CorrealeCarmine OstacoloMichele GallaminiCarole A. PaleyMichele LuchettiCarolin MenzelMichele MariCarolyn DewaMiguel Angel Gonzales GayCarsten PosovszkyMihai MusteataCaterina AnaniaMihir Dilip WechalekarCatherine MunnsMikhail V. VasinCecilia M. PrêleMilan TerzicCees A. SwenneMilena JankowskaCelestino SarduMiltiadis KrokidisCezary WojcikMimily HarsonoChad CarrollMing ZhangChandima P. KarunanayakeMin-Seong HaChandni HindochaMirela FrandesChangxiang WangMirela HabibovicChao ChenMirjana TurkaljChao PengMirko ManettiChao-Hung KuoMirna ŽulecCharat ThongprayoonMirza PojskićCharles W. LanksMithilesh Kumar JhaCheng-Chieh YenMitsugu HachisuCheng-Fang YenMoacir MarocoloChia-Chun ChiangMohamad M. SaabChiara CarlomagnoMohamad ParnianpourChiara CiccareseMohamed AshryChiara GiraudoMohamed HassanChiara LazzeriMohammad Harun RashidChiara LoriniMohammad Zakir HossainChien-Hsieh ChiangMohammed RuziehChien-Ming ChaoMohan RudrappaChih Chung ChenMoises Rodriguez-GonzalezChih-Yen ChenMojtaba KavianiChristiaan MooijMollie RubenChristian GorzelannyMonica BotelhoChristian SchulteMonica GalloChristine RondaninoMonica K TorstveitChristoph SpangMonika HertenChristopher NetschMonika SzeligaChristopher TaberMonish Ram MakenaChristos TikellisMuna J. TahirChungChun WuMuskan KukrejaChung-Hwan ChenMustafa AtarChunhuan LaoMuthukumar KannanChun-Hung SuMyrella PaschaliChun-Jung ChenMyriam Guerra-BalicChunxiao LiNagarjuna Reddy CheemarlaCidália Dionísio PereiraNakamura TaishiCiro AbbondanzaNaoki IwamotoClaire CeriniNarendar Claire TournyNarothama Reddy AeddulaClaudia AlessandriNaser AlsharairiClaudia BrognaNasser AlamiriClaudia PisanuNatalia Balague SerreCláudia VazNatalia DrabińskaClaudio RenziNatalia Nowacka-JechalkeClayton L. CamicNatalia Ukleja-SokołowskaClyde WilliamsNatascia RinaldoColin EslerNathalia Fernandes FagundesColleen A ThomaNaufal ZagidullinCorina Marilena CristacheNaveen SinghCornelis SierNazita TaghaviCorsino Rey GalanNeeraj ChauhanCostantino BalestraNehalennia Van HanegemCostas FourtounasNeil ClarkeCourtney KippsNeil E. FowlerCrescenzio SciosciaNicholas BradshawCrisanta-Alina MazilescuNicholas J. HansonCristian FioriNicholas PetersenCristina CarreteroNicola Luigi BragazziCristina M. FaillaNicola MumoliCristoforo PomaraNicola Zampieri Daiane G. FrancoNicolás Ruiz-RobledilloDale QuestNicole GervaisDamian ShinNikola StambukDana BadauNimer MurshidDaniel AlbaughNimrat ChatterjeeDaniel Almeida MarinhoNitin KumarDaniel Alonso-AlconadaNoel GibneyDaniel HackettNorbert Joachim TripoltDaniel J. LamportNour BaïzDaniel J. ShiwarskiNuruddin UnchwaniwalaDaniel JohnsonOctavio CabaDaniel López-LópezOhad RonenDaniel R. MachinOlaf DietrichDaniela Acquadro MaranOlga M PulidoDaniela MartiniOlga RiklikieneDaniele VanniOlga ScudieroDaria ChmielewskaOlivier P. TraxerDario de BiaseOmesh ToolsieDario Di StasioOnur TürkoğluDario SiniscalcoOsama A. TashaniDariusz BoguszewskiOto HanušDavid BellarOtto MayerJr.David CromptonOu BaiDavid DrutovicPablo Cervera-GarviDavid G. McIntoshPablo Martinez-AmezcuaDavid Gómez-AndrésPalanisamy NallasamyDavid M. DudzinskiPalmiro CornettaDavid ManninoPanagiotis PeitsidisDavid MorrisPanagiotis TsaklisDavid N NaumannPanayiotis D. MegaloikonomosDavid PejoskiPantelis T. NikolaidisDavid RosenthalPaola FerrariDavid StephensenPaola IratoDavid ZahriehPaola SavoiaDavid ZweikerPaolo CaimiDavide Giuseppe RibaldonePaolo ColombaDavide SustaPaolo SeverinoDa-Wen LuParine NRDebra A. TristramPartha RayDejan NikolicPasca Denis GrisPascal PineauDenis I. BurchakovPasquale CianciDenis KainovPasquale ParibelloDespina D. BrianaPasqualina LaganàDevasmita ChoudhuryPatrice ForgetDhananjay YadavPatricia SchofieldDhanapal GovindarajPatrick LegembreDhelal Al-RudainyPatrizia GiannoniDiane ClarkPaul B. NolanDiego RaimondoPaul ConstableDimitra DimitrellouPaul M. VespaDimitra VangeliPaul ZarogoulidisDimitri PoddighePaula ChavesDimitrios DionysopoulosPauline GulliverDimitrios I. AnyfantakisPawel MiotlaDimitrios P. BogdanosPaweł Petkow-DimitrowDimitris FilippiadisPayam BehzadiDina Di GiacomoPedro AciénDinesh GundapaneniPedro MorouçoDinesh JillellaPedro MunueraDinesh ThummuriPegah KhoshpouriDipesh DhakalPei Ling ChooDivya RamchandaniPei-Fung WuDiyana V. IvanovaPengfei XuDjuro KosanovicPeter D. WongDolores MarrodanPeter DarlingtonDomenico Azarnia TehranPeter De LeeuwDomenico SantoroPeter E. MurrayDomenico SergiPeter HosickDominic HarmonPeter JarcuskaDong In SuhPeter MacIntoshDongshan ZhuPeter P RainerDong-Zong HungPeter SchemmerDorAnne M. DoneskyPeter WesterholmDorota FormanowiczPhillip Anthony MooreDorothy TeegardenPiercarlo ParigiDorte Bek FolkvardsenPierre SiéDouglas DunlopPietro GentileDragana M. StanojevicPietro VajroDubravka Švob ŠtracPiia KarisolaDunja Maria Baston-BüstPilar MatudDuo ZhangPilar Puertas MoleroDustin Scott KehlerPing SongEbtihal AlshabibPing ZouEddy KruegerPingfeng ZhangEden Morales-NarváezPin-Kuei FuEdinson Dante Meregildo RodriguezPiyush BaindaraEdita JankauskienėPlachouras DiamantisEdith ClarosPontus HenrikssonEdoardo RaposioPooja TeotiaEdoardo StaderiniPouya SaeediEduard KurzPragney DemeEduardo Garcia-RicoPrasanth GovindanEdward FreemanPrashant SakharkarEdward GorhamPrzemysława Jarosz-ChobotEdward N. HarrisPuneet BajajEdward RileyQin Xiang NgEiichi WatanabeRabin GyawaliEinar ArnbjörnssonRachel DesailloudElena Constanta AmăricăiRaffaele GiordanoElena D BazhanovaRaja MuthupillaiElena ToschiRajeev AuroraElena TsourdiRajesh BotchuElena V. TchetinaRajesh Kumar GandhirajanEleonora NuceraRajeshwary GhoshElisa CairrãoRajib MukherjeeElisabetta BalestroRalf EberhardtElisabetta BianchiniRam JagannathanElisabetta MerigoRamesh WijayaEliza NelsonRami A. Al-HoraniElizabeta B. Mukaetova-LadinskaRamūnas UnikasElizabeth Nappi CorrêaRaphael BorieElizabeth VafiadakiRaphäel P.H. MeierElsa Frideborg RonningstamRaphaele De la TorreElzbieta Jarocka-CyrtaRasa BarkauskienėElżbieta MillerRasmus RiviniusEmad Al-DujailiRaúl Ferrer PeñaEmanuel VamanuRavinder KaundalEmanuela SalzanoRavinder MalikEmil ComanRavirajsinh JadejaEmiliano AntigaRebecca HobanEmilio SaccoRegina Fölster-HolstEmmanuel BonneyRiccardo FerraciniEncarnación G. CarreteroRiccardo MastroianniEnrique OrtegaRiccardo SarzaniEnzo IerardiRicha SoodEnzo Maria VingoloRichard KonesEric BravermanRichard LassEric Santoni-RugiuRichard Steven PappasErik KarlssonRickard NordénErin McAllumRie Eriksen BechsgaardErnesto LimaRimantas KodziusEugenia AllegraRimtautas GudasEugenia VlachouRinaldo PellicanoEugenio CavalliRittu HingoraniEva WillaertRob SiebersEvgenia A. GourgariRobail YasrabEwa KędzierskaRobert BuckiEwa PiątkowskaRobert GaudinEwa WolińskaRobert SmolicEwelina DziurkowskaRoberta CondoFabien ForestRoberta ImperatoreFabio CoppedèRoberto Farina De AlmeidaFabiola Moutinho Roberto AiolfiFabrizio LuppiRoberto ScandurraFadi BadlissiRobin M. Voigt-ZuwalaFatih DemirRobyn Laura KosinskyFazal-ur-Rehman BhattiRocco SpagnuoloFederica ColliniRodrigo ValenzuelaFederica PapaccioRoger C. HoFederico BiscettiRoger Ho CMFederico UsuelliRohitash JamwalFelice PirozziRoland KrämerFelice SiricoRolf Alexander JanosiFélix Zurita OrtegaRoman F. BošnjakFeng GuRoman LeischikFeng HeRomeo PatiniFengjie SunRonan MurphyFerenc IhaszRosa MendezFergus GuppyRosa PilolliFernando AndradeRosa RoyFilipa F. ValeRosa SammarcoFilippo RenòRosalba SiracusaFilippo TorielloRosan W MeyerFlavio Tarasoutchi TarasoutchiRosaria M. RuggeriFlorin OanceaRosaria OrlandiFoteini MalliRosemary HiscockFrancesca AndreoniRositsa Denkova-KostovaFrancesca DuraturoRossella CannarellaFrancesca SaladiniRoxana Maria NemeșFrancesco CiconeRozeta SokouFrancesco De FrancescoRubén F. González LaredoFrancesco LocatelliRubén Sánchez-GómezFrancesco TovoliRui MatosFrancis DumlerRyan McGrathFrancisco Cabrera-ChávezS. J. ZaidiFrancisco Cavas-MartínezSabine Sator-KatzenschlagerFrancisco Herrera-GómezSadaf SharfaeiFrederick KaskelSaeed JerbanFumiaki FujibeSaeed SadeghiGábor MátyásSaleky García-GómezGabriel Claudiu TenderSalil LachkeGabriela TopaSalim SuraniGabriele CarulloSalvatore Giovanni VitaleGabriele Di CarloSam SchulmanGabriele MascheriniSamir DwidmutheGadi BorkowSamson N. DowlandGaetano IsolaSamuel MabbottGareth WaltersSamuel WinerGary HooperSamuli JaakkolaGaurav GhagSamy A. AzerGavin LambertSandra LejnieceGeorge AdonakisSandro GelsominoGeorge R. MatcukSangeeta KhareGeorgia RagiaSangram SamalGeorgian BadicuSanja AveicGerald ChiSara C. ZapicoGergely VargaSara FalsiniGeri McLeodSara GioiaGhose BishwajitSara MantiGiancarlo GarutiSarah E. GullbrandGianluca SerafiniSarah J. HemleyGianluca TestaSarah KendalGilliard LachSarah MeyerGina MandaSassan HafiziGiorgio ScivolettoSatoru KobayashiGiovanna GraziadeiSatoshi MorimotoGiovanni CimminoSatoshi YanagisawaGiovanni MerlinoSaulius RočkaGiovanni MisciagnaSaverio GnudiGiovanni RaffaSayyed Ali SamadiGiovanni TarantinoSebastián MondacaGiulia SpoletiniSebastiano MassaroGiuseppe LosurdoSeiji ShiotaGiuseppe MandraffinoSeitaro NomuraGiuseppe TroianoSeon-Yong JeongGiuseppina AndreottiSerge BrandGloria ArriagadaSergio Cuevas-CovarrubiasGoh OhjiSerkan YılmazGopika G. NairSeung-Yup KuGrace FarhatSeyed Amir Danesh-SaniGrażyna Czaja-BulsaShahid MansuriGrażyna JanikowskaShailendra MauryaGregory ConnersShalaka Ajit MulherkarGretchen E TietjenShankar MunusamyGrzegorz JakielShannon NowotarskiGrzegorz NowickiSharon InmanGudrun DiebergSheikh M. AlifGudrun KunstShelly LuGuglielmo DurantiShervin AssariGuida Da PonteShigeo TakebayashiGuillermo A. Cañadas-De La FuenteShih-Kuo ChenGuiting LinShilpi VergheseGunjan AroraShinya SuzukiGunther HempelShiva RadhakrishnanGuozheng WangShivang DesaiGuruswamy MaheshShow-Mei ChuangGwénolé LoasShubhi KaushikGwyn LewisShu-Chun ChuangGyula AcsadiShukun ChenHadi MessihaSigita BurokienėHailong SongSigrid Bjerge GribsholtHaizal HussainiSijan BasnetHamid SuhailSilvia BielsaHan KemperSílvia Cervero-AragóHanna Gendek-KubiakSilvia Izquierdo ÁlvarezHannu LuomajokiSilvia Laura BoselloHans F. FuchsSilviu BrillHanumantha Rao MadalaSimon ChiuHaralds PlaudisSimona FaraciHarold L. KennedySimona Gerocarni NappoHaroon MajeedSimona LattanziHaruki HoikeSimone CheliHarvey BurnettSimone FerreroHarvey CheckowaySimone GarzonHassane ZouhalSimone VidaleHazel PartingtonSizhao LuHector Beltrán AlacreySlavko RoganHelen C. GallagherSławomira Drzymała-CzyżHelen HuSoenke BoettgerHelena FelgueirasSolvejg L. HansenHelena SoaresSomashekar G. KrishnaHenrik HorwitzSomasundaram PrasadhHideaki OkajimaSomchai AmornyotinHie-Won HannSonja KlebeHilal ZaidSonsoles InfanteHilde KelchtermansSophie LelorainHirokazu MadokoroSorin HostiucHiromasa HasegawaSoumyadeep DeyHiroshi IchikawaSpyridon MethenitisHiroshi KurokiSpyridoula MarakaHiroshi YaoSrinivas SubramanianHirotaka MiyamotoStacey SchutteHiroyuki MatsubayashiStavros J. BaloyannisHolger Karl H. TillStefan HeberHouria DaimiStefan LautenbacherHoward E. StrasslerStefan Michał AnzelewiczHoward KirshnerStefan NaydenovHsin-Fang ChungStefan NilssonHui Jai LeeStefan ReersHui-Ting LinStefan W. VetterHung-Lung ChiangStefano ComaiHung-Yi ChuangStefano MinistriniHuseyin BayazitStefano PaganoHyang Mi KoStephanie L. OsbornHyosun ChoStephanie PlummerHyun-Soo ChoStephanie ShermanIan CulpanStephen A. FletcherIan NewhouseStephen R WelchIbrahim FaruqiStephney WhillierIbrahim HassanSteven E. WilliamsIftíhar KöksalSteven G. GrayIgor AlbizuaStuart F. QuanIgor GruićSu SongIgor ŁoniewskiSubash HeraganahallyIgor SplichalSudheer RavuriIlias KarametosSudhir BaswanIlona KopytaSueli Coelho Da Silva CarneiroIn Sik YunSumeet SolankiInah KimSuneel KumarIndu B. AhluwaliaSunil KumarInes KožuhSuresh SamsonInes MármolSusan LewisInês PáduaSusana LozanoInês Pereira De FigueiredoSusana SecaInger Lise GadeSuvara K. WattanapitayakulIngrīda Rumba-RozenfeldeSyed Haris OmarIoan Stefan FlorianTadas LatkauskasIoana MozosTahsin OzpolatIole VozzaTaina T. NieminenIonel BondocTakahiro KannoIori SakakibaraTakahiro YoshidaIppokratis MessaritakisTakeshi HisadaIrene FrischaufTakeshi KatayamaIrina KontsevayaTamar KinaciyanIris CaramalhoTamara GulicIris ReuterTan Boon TohIsao OtsukaTanja Grubić KezeleIsha SharmaTanja PejovicIsmaeel YunusaTara McmorrowIstvan AntalTareq SalehIstván RuzsicsTatsuya MoriyamaI-Te LeeTeet SeeneIulia-Diana MuraruTe-Huei YehIva KiracTeodora Alexa-StratulatIván GuerraTeresa Mary MacDonaldIvan Malagoli LanzoniTeruhiko ImamuraIvan MiskulinTerumi Midoro-HoriutiIvan VargaTetsuji FujitaIveta MintāleTetsuo NishikawaIvica BlazevicTetsuya YumotoIzabela GrzegorczykThadeus TrusIzabela ZakrockaThayse Natacha GomesJacob SellarésThi-Huong NguyenJae-Bum JunThinh NguyenJames Kumi-DiakaThomas G. PowerJames O’BeirneThomas LambertJames R. FuchsThomas MeinelJames TsaiTiago JacintoJames X. HartmannTian TianJamie S. ShethTilo NiemannJan BilskiTim KilnerJan Jaap H.M. ErwichTim SciallaJanet RimmerTimothy A. DamronJanet S. CarpenterTing-Feng WuJanhavi NagwekarTirupapuliyur DamodaranJanin HenkelTiziana LarussaJan-Ulrik DahlTobias W. SinnbergJared G SmithTom Harald EdnaJarmila CelakovskaTomas LapinskasJar-Yi HoTOMASZ M KARPIŃSKIJason AuTomasz OlesińskiJavier Mateu-de AntonioTomasz PaszkowskiJaw-Yuan WangTomasz PowrózekJayanta Kumar PatraTomoaki TakataJayanthi ManiamTomoki KoshoJean A. BoutinToshinobu MiyamotoJean AmiralToshiyuki KawaiJeanine YoungTracy DeWaldJean-Michel LecerfTrevor BarssJean-Paul StahlTsung-Chien LuJean-Pierre BaeyensTsung-Hsueh LuJeffrey RothschildTsvetelina VelikovaJennifer BorowskyTudor Lucian PopJennifer HaynesUdo JeschkeJennifer R. SchroederUlf NestlerJennifer WambachUlrike Nowak-GöttlJenny WilkinsonUmang SwamiJer-Ming ChangUmile Giuseppe LongoJerzy RomaszkoUmit CetinJesse Upasana ShokalJessica HolmgrenUrvish PatelJesus M. Lopez RodillaVaidotas MarozasJia LiuValaree WilliamsJiafen HuValentina ColistroJiajia XueValentina LiakinaJian ShiValeria ContiJie (Jane) WangValérie HoxJimmy T. EfirdVânia Canterucci Gomide-ÇakmakJiney JoseVanja TadićJing LuVaratharaj MounasamyJin-Ming WuVasanth KumarJisun PaikVasanth StalinJiyun ZuVasileios KouranosJoan OlivaVasileios StavrouJoan Ramon TorrellaVasily KashtalapJoan Torras AmbrosVassilis AthanasiadisJoana CostaVenkateswara Reddy GogulamudiJoanna DrzeżdżonVenugopal GangurJoanna KarolkiewiczVeronika AleksandrovychJoanna Myszkowska-RyciakVeronika NollJoanna RogVeronika ŽižkováJoanna RoszakVesna BabicJoanna WietrzykVicente Seco-RoviraJoanne Van RynVicky WangJoaquim M. B. PinheiroVictor BlümlJohan EngdahlVíctor García-GonzálezJohannes MayrVíctor M. RiveraJohn A. BurgessVictoria H. J. RobertsJohn FrancisVictoria ZiesenitzJohn K TriantafillidisVijayalaxmi GuptaJohn L. McIntoshVikrant RaiJohn PaigeVincent ChungJohn T HeikerVincenzo ScuderiJohn W. FrewVincenzo TufarelliJolanta LewkoVirendra ChaudhriJolanta Orzelska-GórkaVishal MaliJon SenVishal SinghJonathan S. TamViswanath RagupathyJone A. StanleyVito D’AndreaJoo Hyoung LeeVivian AuyeungJoohyun RheeVladimir I. GridnevJoonas H KauppilaVsevolod KonstantinovJordi Pérez TurVucenik IvanaJorge Santos-JuanesWakako TakabeJos BloemersWalter L. StrohmaierJosé A. OteoWegene T. BorenaJOSE ANTONIO DE PAZWei LeiJosé Antonio Morales-GonzálezWeiguang YangJosé Carlos TavaresWeimin GuoJosé De Jesús Sandoval-PalomaresWenbin TanJose H MarcoWen-Chi ChouJose L. Flores-GuerreroWenqing YinJose Luis UbagoWenyen JuanJosé Manuel FerroWerner J. D. OuwendijkJose Manuel LozanoWesley KephartJosé Pedro Cerón-CarrascoWilhelm MistiaenJosé SilvaWilliam HorsnbyJosé SuazoWilliam R. ReedJosé TuellsWioletta Adamus-BiałekJosef YayanWissem MnifJoseph GovanWon BaeJoseph O. Falkinham IIIWoon-Man KungJosephine StorekWouter KokJoshua E. MuscatWycliffe W.Simiyu NjororaiJoshua EverhartXavier SantamaríaJoshua ZadroXia XiaoJosip A. BorovacXiangmin LvJoško MarkićXiao YangJoško OsredkarXiaoyan ChenJoyce ThompsonXingjuan ChenJózsef KónyaXudong HuangJuan Aníbal González-RiveraYael CaspiJuan Gómez-SalgadoYagmur OzturkJuan JovelYa-Ju ChangJuan Miguel Martínez-GalianoYale Tung-ChenJudit DanisYang CaoJudith WeinerYang ChenJuei-Tang ChengYang ZhaoJuliann M. Di FioreYaniv DotanJulie CampbellYasumasa KatoJulio Plaza-DíazYen-Ko LinJun LuYerim KimJun UdagawaYet Hong KhorJune Young ChoiYi SunJunfei XiaYi-Jen LiaoJungsun LeeYijie WangJūratė MacijauskienėYijun ZhangJurgita GailiteYing-Jie PengJuscelino TovarYingyue ZhangJustyna GodosYino WuJwu-Lai YehYiqiang ZhanJyothi MenonYi-Ting LinKaitlin DayYixiang ZhangKalpana NathanYixuan ZhangKamil FijorekYoh AritaKamonpun UssavarungsiYohannes Woubishet WoldeamanuelKar Wey YongYon-Cheong WongKaren FrancisYong Jin KimKaren NagelYonggeun HongKarl AndriessenYoona KimKarl KozlowskiYosra A. HelmyKatarzyna Jochymczyk-WoźniakYouness RioualiKatarzyna KotfisYu KoyamaKatarzyna PawlakYu-Chao ChangKatarzyna WiktorskaYueh-Ying HanKatharina KusejkoYuexin XuKatharine HardingYuichiro HayashiKathrin WunschYuji NagayamaKatia Candido CarvalhoYu-Lueng ShihKatja UpadyayaYunfei LiKatsuhiro MasagoYuri BattagliaKazuhiko KotaniYves MILLEMANNKazuhiro P. IzawaYvonne DanielKazuki IdeZachary StrombergKazunori NagasakaZackary CiconeKedra WallaceZahra BazrafshanKeiichi IshidaŻaneta Kimber-TrojnarKeiichi MatsubaraZbigniew BartuziKeisuke KuwaharaZebekakis PantelisKelli SasZeljka VanicKelly-Ann BowlesZeneng WangKensuke KumeZhaohui XiongKensuke MatsushitaZheng Feei MaKenya YamanakaZhihong YuanKeun-Yeong JeongZhonghua SunKevin ChenZhongwei LiuKevin KooZhuo XingKhalid El BairiZoe KopsaftisKhalil N. BitarZorita DiaconeasaKibwei A McKinneyZsofia LazarKiran K. UpadhyayZygmunt WarzechaKiwako Yamamoto-Hanada


